# Retropharyngeal Calcific Tendonitis Mimics a Retropharyngeal Abscess

**DOI:** 10.1155/2013/818561

**Published:** 2013-07-15

**Authors:** Natasha Pollak, Sonya Wexler

**Affiliations:** Department of Otolaryngology—Head & Neck Surgery, Temple University School of Medicine, 3440 North Broad Street, Kresge West, Philadelphia, PA 19140, USA

## Abstract

Retropharyngeal calcific tendonitis (RCT) is an uncommon, self-limiting condition that is often omitted in the differential diagnosis of a retropharyngeal fluid collection. This condition mimics a retropharyngeal abscess and should be considered when evaluating a fluid collection in the retropharyngeal space. Although calcific tendonitis at other sites has been well described in the medical literature, it appears that this entity has been underreported in the otolaryngology literature where only a few case reports have been identified. Presumably, the actual incidence is higher than the reported incidence, due to lack of familiarity with this disorder. As an otolaryngologist's scope of practice includes the managements of retropharyngeal lesions, it is important for the otolaryngologist to recognize the presentation of acute RCT and be familiar with appropriate treatment strategies. Retropharyngeal calcific tendonitis presents with neck pain, limitation of neck range of motion and includes inflammation, calcifications, and a sterile effusion within the longus colli muscle. Treatment is medical with nonsteroidal anti-inflammatory medications. RCT does not require surgical treatment, and an accurate diagnosis can prevent unnecessary attempts at operative drainage. In this study, we discuss two cases of RCT, summarize the salient features in diagnosis, including key radiologic features, discuss treatment options, and review the literature.

## 1. Introduction

We present two cases in which the initial presentation was suggestive of a retropharyngeal abscess, but upon further review, both patients were ultimately diagnosed with retropharyngeal calcific tendonitis, an inflammatory, rather than infectious, condition. The purpose of this case series is to raise awareness of retropharyngeal calcific tendonitis (RCT) as an unusual but possibly not rare cause of a retropharyngeal fluid collection, as well as highlight the salient diagnostic features that will allow the surgeon to make the correct diagnosis, avoid unnecessary incision and drainage, and commence effective treatment early in the disease course. It is important to be able to differentiate between retropharyngeal tendonitis and an abscess, as this determines the correct course of treatment.

## 2. Case Presentation #1

A 37-year-old man presented to the emergency room with a chief complaint of neck pain and sore throat. He first noticed the neck pain 3 days earlier, but did not think much of it. The pain gradually worsened to 10/10 on the 0–10 pain scale. The neck pain was exacerbated by head movement and swallowing, but ameliorated by laying supine. The pain did not radiate to his upper extremities. He also complained of throat and neck swelling as well as right otalgia. He denied fevers or chills. His only sick contact had been his daughter with streptococcal pharyngitis 1 week earlier. Of note, he also denied traumatic injury, photophobia, cough, rhinorrhea, dysphagia, hoarseness, or shortness of breath. The patient received clindamycin and dexamethasone in the emergency room. 

On examination, the patient was alert, oriented, and nontoxic appearing but clearly uncomfortable. He was afebrile and his vital signs were within normal limits. He was not stridorous or drooling. There was a limited range of motion due to pain that was localized to the suboccipital neck. This area was minimally tender to palpation as well, but there were no signs of external trauma or lymphadenopathy. Examination of the eyes, ears, nose, oral cavity, and cranial nerves was within normal limits. Examination of the oropharynx showed erythema but no exudate or bulge to suggest an abscess. A flexible fiberoptic nasopharyngoscopy showed hypertrophy and erythema of the adenoid pad but was otherwise normal.

Laboratory testing showed a leukocyte count of 8,600 on admission, and there was no left shift. Throat culture and blood cultures were negative.

**Figure 1:**
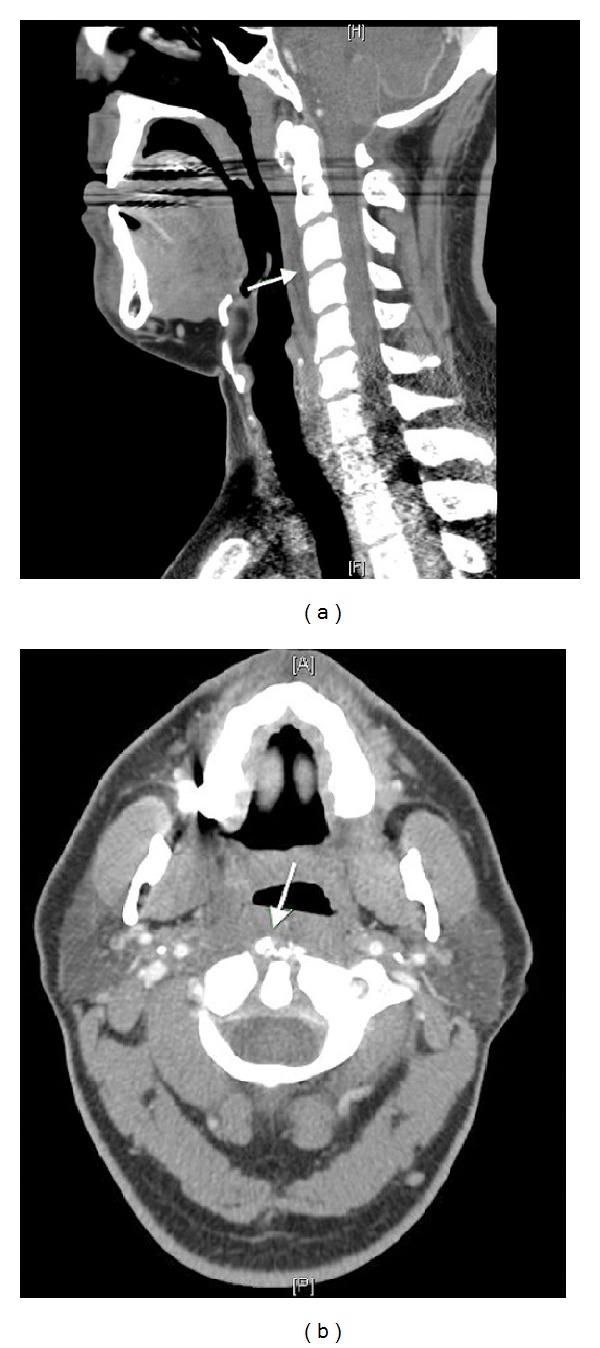
(a) Sagittal CT neck with IV contrast shows a prevertebral fluid collection (arrows). (b) Axial CT neck with calcifications of the longus coli tendon (arrow).

Prior to admission, the emergency doctor obtained a computed tomography with intravenous contrast of the neck which was consistent with a noncontrast enhancing oval-shaped hypodensity of the retropharyngeal space extending from the C1-C2 level to the C5-C6 level. This lesion measured 8.2 cm in length with a cross-sectional diameter of 3.0 × 1.3 cm (Figure 1). Based upon the symptoms, physical exam, and CT findings, the patient was started on intravenous antibiotics.

**Figure 2:**
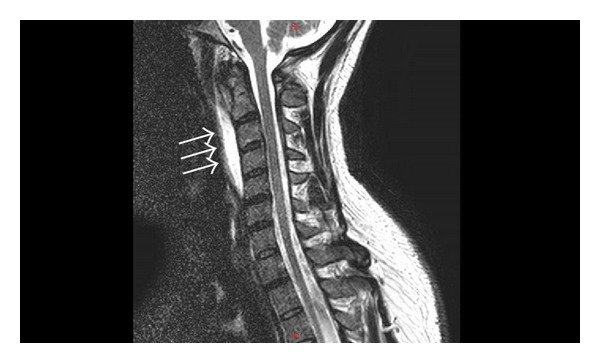
Sagittal T2-weighted MRI of the neck shows bright signal representing a prevertebral fluid collection.

On hospital day #2, magnetic resonance imaging of the cervical spine was obtained. The MRI was consistent with a retropharyngeal abscess from level C1 to C5 (Figure 2). Also, the leukocyte count had increased to 14,200, still without a left shift. Despite these findings, the patient appeared to be clinically improving. Neck range of motion improved, odynophagia slowly improved, and he remained afebrile. The otolaryngology team discussed with the patient surgical incision and drainage of the presumed retropharyngeal abscess and the need for surgical incision and drainage if he began to clinically decline. The patient remained clinically stable on amoxicillin + sulbactam, Flexeril, and morphine. His leukocyte count decreased to 9,600 by hospital day #3.

**Figure 3:**
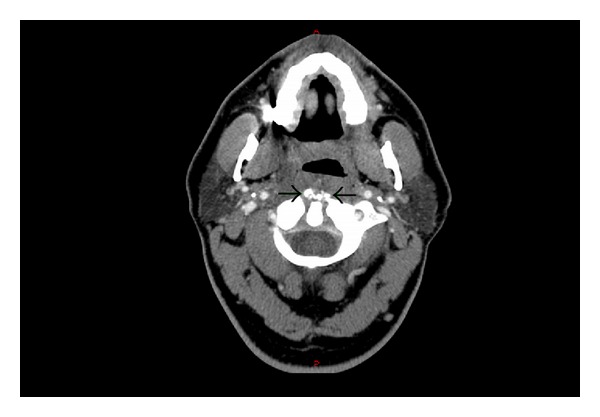
Axial CT of the neck with IV contrast on hospital day #4. Calcifications of the longus coli tendon are apparent (arrows).

On hospital day #4, CT of the neck was repeated considering the difference in the prior CT and MRI findings and the clinical improvement of the patient. The repeat CT scan showed calcifications at the attachment of the longus coli tendon at the C1-C2 level with a retropharyngeal effusion that had improved slightly since the prior study. No evidence of abscess was seen. Inflammatory changes of the tonsils, adenoids, and cervical lymph nodes were identified (Figure 3). This supported our decision not to perform an incision and drainage.

Based on the history, physical examination, and radiographic findings, the most likely diagnosis at this point was acute calcific tendonitis of the longus coli muscle. The patient was started on a nonsteroidal anti-inflammatory medication (NSAID) and was also discharged with a seven-day course of antibiotics. Approximately three weeks after discharge, he was doing well, with complete resolution of neck pain and pharyngitis.

## 3. Case Presentation #2

A 69-year-old woman presented to the emergency room with severe neck pain and sore throat for two days. Her pain was dull and diffuse, radiating to the front of her neck. The pain was exacerbated by swallowing and improved with rest. She was also experiencing dysphagia, hoarseness, and limited range of motion of her neck due to pain. She denied fevers, traumatic injury, or known sick contacts. The patient was treated with IV clindamycin and dexamethasone in the emergency department with some improvement in her symptoms.

The patient's past medical history was significant for remote tonsillectomy. She was taking warfarin for atrial fibrillation and atenolol for hypertension. She was allergic to penicillins. She was a prior smoker with a 100 pack-year smoking history, but denied drinking alcohol or using illicit drugs. Her family history was noncontributory.

On examination, the patient was nontoxic appearing and afebrile. There was no sialorrhea and her breathing was unlabored. Range of motion of her neck was limited. There were no oropharyngeal exudates, retropharyngeal edema, or erythema. Examination of the eyes, ears, nose, oral cavity, and cranial nerves was within normal limits. We performed a fiberoptic laryngoscopy. The nasopharynx, oropharynx, and larynx were normal.

Laboratory testing showed a leukocyte count of 11,800 on admission. She was admitted to the medical ward, and IV clindamycin was continued.

**Figure 4:**
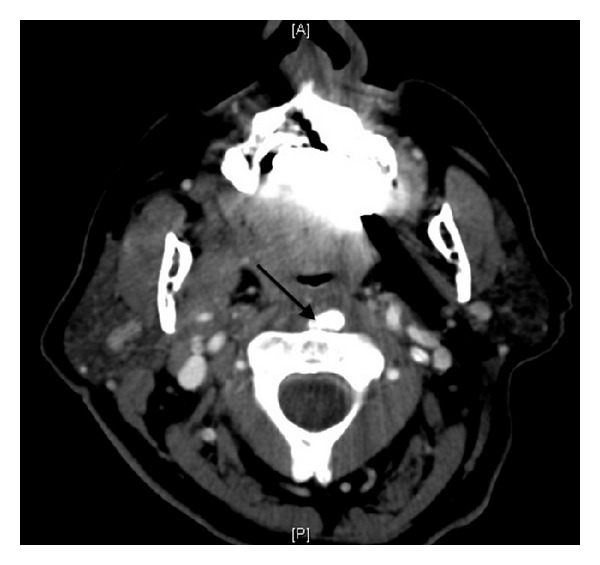
Axial CT of the neck with IV contrast shows calcifications anterior to the body of C2 (arrow).

CT neck with intravenous contrast showed diffuse thickening of the prevertebral soft tissues. There was a calcific deposit anterior to the distal end of the body of C2 which was within the soft tissue thickening at the level of the oropharynx (Figure 4). Considering the CT findings and her clinical picture, the diagnosis of acute calcific tendonitis of the longus coli muscle was favored. At that point, she was started on a nonsteroidal anti-inflammatory medication (NSAID). After only 24 hours of medical therapy, the patient already noted a vast improvement in her neck pain, and she was cleared for discharge. Clindamycin was discontinued. The patient was discharged on ibuprofen for 10 days. Because the patient was also taking warfarin, it was recommended that she follow up with her primary care doctor within a few days to check her coagulation parameters with the addition of ibuprofen.

Based upon her physical exam and radiographic findings, it was clear that the cause of the retropharyngeal fluid collection was not an infectious etiology, but rather acute calcific tendonitis of the longus coli muscle became the working diagnosis. Additionally, the patient's quick response to treatment with an NSAID supported this diagnosis.

## 4. Discussion

Acute retropharyngeal calcific tendonitis of the longus coli muscle is an uncommon disorder and as such is often overlooked by physicians. While there are several case series in the emergency medicine and the radiology literature, this entity is relatively poorly described in the otolaryngology literature. Presumably, the incidence of RCT is underreported in otolaryngology due to the lack of familiarity with the disorder.

The longus coli is a paired thin flexor muscle that spans the anterior surface of the vertebral bodies from C2 and T3. It derives its blood supply from the vertebral, inferior thyroid and ascending pharyngeal arteries and is innervated by the second through sixth cervical spinal nerves [1]. The deep layer of the deep cervical fascia lies anterior to the muscle and posterior to the retropharyngeal space.

As first described by Hartley in 1964, calcium deposits in this region have been associated with symptoms of decreased range of motion on the neck, odynophagia, dysphagia, and throat swelling [2]. As with deposition of calcium within joint spaces, there are associated inflammatory changes, which, in the case of calcium deposition within the longus colli muscle, is associated with tendonitis.

As the otolaryngologist is often called to determine the treatment plan for patients with retropharyngeal fluid collections, the otolaryngologist needs to be familiar with the clinical presentation of RCT and be able to differentiate it from an abscess.

Ring and colleagues in 1994 described a series of 5 patients, one of whom had an open biopsy for suspected malignancy. When this tissue was examined microscopically, an inflammatory foreign body response was found, likely in response to hydroxyapatite deposition [3]. This has been the widely accepted pathophysiologic mechanism of longus coli tendonitis. They also reported edema in the posterior aspect of the nasopharynx. On flexible fiberoptic exam, our male patient did appear to have inflammation and erythema of the adenoid pad which had not been previously identified. This is likely due to spread of inflammation along the fascial plane between the adenoids and longus colli muscles.

The differential diagnosis of a retropharyngeal fluid collection on imaging is extensive, and the edema associated with tendonitis can be confused with a retropharyngeal abscess. Pathognomonic radiologic findings in RCT are the presence of a prevertebral effusion from C1 to C4 and calcifications below the anterior arch of C1. In some instances, calcifications are not found and a contrast CT is warranted to verify whether the fluid collection is ring enhancing [4]. Both of our patients had a calcific deposit along the longus colli muscle as well as a retropharyngeal effusion. More recent literature also reports that calcifications at C4-C5 could be associated with symptoms of respiratory distress [5].

Even though retropharyngeal calcific tendonitis is a self-limiting condition, patients do seem to improve rapidly with administration of NSAIDs. Both of our patients showed significant improvement with NSAIDs, although they were also initially treated with a limited course of antibiotics which was likely excessive [6]. Importantly, the otolaryngologist should review the CT with the radiologist as the radiologist often has limited clinical information about the patient. Often, if the history consists of upper respiratory tract infection symptoms with neck pain and dysphagia and there is a retropharyngeal fluid collection, the most common etiology is infectious. However, the otolaryngologist should be aware of RCT and consider including it in the differential diagnosis. Once RCT is diagnosed, NSAIDs should be started without delay.

Otolaryngologists should be familiar with the entity of retropharyngeal calcific tendonitis as it may not be as rare as previously reported. As otolaryngologists become more familiar with retropharyngeal calcific tendonitis, clinical presentation, and typical radiologic findings, unnecessary attempts at incision and drainage can be avoided.
